# Neurobiology of Sleep Disturbances in PTSD Patients and Traumatized Controls: MRI and SPECT Findings

**DOI:** 10.3389/fpsyt.2015.00134

**Published:** 2015-09-28

**Authors:** Davide Nardo, Göran Högberg, Cathrine Jonsson, Hans Jacobsson, Tore Hällström, Marco Pagani

**Affiliations:** ^1^Neuroimaging Laboratory, Santa Lucia Foundation, Rome, Italy; ^2^Division of Psychiatry, Department of Clinical Neuroscience, Karolinska Institutet, Stockholm, Sweden; ^3^Department of Nuclear Medicine, Karolinska University Hospital, Stockholm, Sweden; ^4^Neuropsychiatric Epidemiology Unit, Institute of Neuroscience and Physiology, Sahlgrenska Academy, University of Gothenburg, Gothenburg, Sweden; ^5^Institute of Cognitive Sciences and Technologies, National Research Council, Rome, Italy

**Keywords:** PTSD, sleep, insomnia, nightmares, voxel-based morphometry, SPECT

## Abstract

**Objective:**

Sleep disturbances such as insomnia and nightmares are core components of post-traumatic stress disorder (PTSD), yet their neurobiological relationship is still largely unknown. We investigated brain alterations related to sleep disturbances in PTSD patients and controls by using both structural and functional neuroimaging techniques.

**Method:**

Thirty-nine subjects either developing (*n* = 21) or not developing (*n* = 18) PTSD underwent magnetic resonance imaging and a symptom-provocation protocol followed by the injection of 99mTc-hexamethylpropyleneamineoxime. Subjects were also tested with diagnostic and self-rating scales on the basis of which a Sleep Disturbances Score (SDS; i.e., amount of insomnia/nightmares) was computed.

**Results:**

Correlations between SDS and gray matter volume (GMV)/regional cerebral blood flow (rCBF) were computed in the whole sample and separately in the PTSD and control groups. In the whole sample, higher sleep disturbances were associated with significantly reduced GMV in amygdala, hippocampus, anterior cingulate, and insula; increased rCBF in midbrain, precuneus, and insula; and decreased rCBF in anterior cingulate. This pattern was substantially confirmed in the PTSD group, but not in controls.

**Conclusion:**

Sleep disturbances are associated with GMV loss in anterior limbic/paralimbic, PTSD-sensitive structures and with functional alterations in regions implicated in rapid eye movement-sleep control, supporting the existence of a link between PTSD and sleep disturbance.

## Introduction

Post-traumatic stress disorder (PTSD) is a condition arising from a psychological trauma involving actual or menacing physical danger to self or others and the experience of acute fear. Symptoms are the involuntary reexperiencing of the trauma in flashbacks and nightmares, distress on exposure to memory triggers, active avoidance of such triggers, and a general numbing of feelings, along with mood and impulse control disturbances like hypervigilance and aggressiveness (1).

Temporary sleep disturbances such as insomnia and nightmares are part of a normal response to trauma (2) and are among the most common symptoms reported by traumatized people (3), whereas long-lasting sleep disturbances are a core feature of PTSD. In fact, in DSM-5, nightmares are comprised under the intrusion symptoms (cluster B) while insomnia under the arousal/reactivity symptoms (cluster E). Insomnia and nightmares are highly prevalent in PTSD patients (4, 5), who report significantly higher rates of sleep disruption when compared with controls (6, 7). Sleep serves a restorative function and facilitates emotional processing; hence, sleep disturbances might intensify and prolong diurnal PTSD symptoms, worsening patients’ ability to recover and contributing to poorer perceived psychological health, also considering that sleeplessness represents a physiological stressor (3, 8). Sleep disturbances have a negative impact on health, functioning, and quality of life while their successful treatment results in more restoring sleep and improvement in these dimensions (9–14).

Sleep disturbances are strong predictors of PTSD. For instance, poor sleep quality and severe sleep disturbances are associated with PTSD severity irrespective of age, gender, psychiatric comorbidity, and type of trauma (15). The presence and severity of insomnia and nightmares within 1 month post-trauma might predict the development of PTSD between 6 and 12 months later (16, 17), whereas increased PTSD symptomatology correlates with reports of more insomnia (18). The presence of trauma-related dreams within 1 month post-trauma is a good predictor of PTSD symptoms severity 6 weeks later (19). Nightmares might predict PTSD symptoms severity independently from insomnia (20) as well as anxiety/depressive symptoms and lower sleep efficiency (21). Finally, nightmares might lead to sleep fragmentation, resulting in increased insomnia severity (22, 23).

In the last two decades, the neurobiology of PTSD have been extensively investigated, and there is now converging evidence that PTSD is typically associated with volumetric reductions in the hippocampus and anterior cingulate cortex [ACC (24–30)] as well as with functional dysregulation in the amygdala and medial prefrontal cortex [including the ACC (31, 32)].

The neurobiological underpinnings of insomnia and nightmares are still largely unknown. Available structural neuroimaging data on insomnia show several limitations and a certain degree of inconsistency (33–35). Some studies converge in pointing at the hippocampus. Bilateral hippocampal volume was found significantly reduced in patients with insomnia (36) and negatively correlated with the duration of insomnia/arousal level (37) or with measures of poor sleep maintenance (38). Consistently, sleep fragmentation and related chronic stress were found to reduce neurogenesis and produce neuronal loss in the hippocampus (39). However, these findings were not replicated by other investigations (35, 40, 41). Structural alterations were also found in the orbitofrontal cortex (40, 42, 43), while larger volumes were reported in the ACC, where insomnia severity correlated with volume (44). Finally, correlations between gray matter (GM) and insomnia severity were also found in the prefrontal cortex (41).

Functional studies have shown that insomniacs show hypoperfusion in the thalamus, basal ganglia, and medial prefrontal cortex (45, 46). Moreover, insomnia severity was found to negatively correlate with CBF in the ACC and insula (47). Finally, resting-state fMRI studies have found that insomniacs show altered patterns of functional connectivity between limbic structures and cortical/subcortical regions (48, 49).

To date, studies investigating brain alterations related to sleep disturbances in PTSD are lacking, with only a few exceptions showing that during REM-sleep PTSD is associated with a hypermetabolism in the brain stem, limbic regions, and basal ganglia (50, 51). Germain et al. (52) have proposed a neurobiological model hypothesizing that sleep mechanisms are major determinants of PTSD, acting via an altered functioning of amygdala and medial prefrontal cortex. On the other hand, a neurocognitive model of nightmares has been proposed, according to which nightmares reflect problems with fear extinction while dreaming and are associated with altered function within a circuitry comprising amygdala, hippocampus, ACC, brain stem, and hypothalamus (53).

The present study aimed at investigating sleep-related brain alterations (i.e., due to the presence and severity of insomnia/nightmares) in PTSD patients and traumatized controls by using both voxel-based morphometry (VBM) and SPECT. On the basis of the reviewed literature, we hypothesize that sleep disturbances will be related to both structural and functional alterations in structures such as amygdala, hippocampus, ACC, and possibly in some sleep-regulating centers such as brain stem, diencephalon, and basal forebrain.

## Materials and Methods

### Subjects

This study is part of a wider research project on PTSD among traumatized employees of the Stockholm public transportation system recruited during the years 1999–2002 [see Ref. (54)]. Thirty-nine subjects were recruited for the present study, either developing (*n* = 21) or not developing (*n* = 18) PTSD. Of these, due to incompatibility reasons, two PTSD subjects underwent only the MRI scanning, while one PTSD and one non-PTSD underwent SPECT only. This implies that for each neuroimaging dataset (MRI and SPECT), analyses were run on 37 subjects, 35 of which were in common. Demographic and clinical characteristics of our sample are summarized in Table 1. When subjects participated in the study, a period of time varying between 8 months and 6 years had elapsed since trauma. Only a full PTSD diagnosis was accepted. Exclusion criteria were a history of psychosis, major depressive disorder and other psychiatric conditions (e.g., bipolar disorder, obsessive–compulsive disorder, and attention-deficit hyperactivity disorder), lifetime or current drug/alcohol abuse or dependency, significant medical condition (e.g., diabetes and tumors), neurological illness, or a history of head injury. In our sample, only three subjects under psychopharmacological treatment (one with SSRI and one with tricyclic antidepressants in the PTSD group; one with SSRI in the non-PTSD group), while none was under psychotherapeutic treatment. The study was conducted in accordance with the Helsinki Declaration, approved by the Ethics and Radiation Safety Committee of the Karolinska Hospital, Stockholm, Sweden, and written informed consent was obtained from all participants.

**Table 1:** **Demographic and clinical characteristics of subjects participating in the study**.

	ALL	PTSD	Non-PTSD	PTSD vs. non-PTSD
*n*	39	21	18	n.s.
Sex (f/m)	12/27	7/14	5/13	n.s.
Trauma type (PUT/A)	28/11	15/6	13/5	n.s.
Psychopharmacological treatment	3	2	1	–
One/more trauma(s)	16/23	3/18	13/5	<0.001
Age	41.2 (8.8)	42.8 (8.7)	39.3 (8.9)	n.s.
SDS	7.6 (3.8)	10.4 (2.8)	4.2 (1.2)	<0.001
HAM-A	26.8 (4.3)	29.7 (3.5)	23.5 (2.3)	<0.001
HAM-D	11.9 (8.1)	17.2 (6.4)	5.7 (4.8)	<0.001
IES	24.3 (19.5)	38.0 (15.2)	8.4 (9.1)	<0.001

*PUT/A, person under train/aggression; SDS, Sleep Disturbances Score (see Materials and Methods); HAM-A, Hamilton Anxiety Rating Scale; HAM-D, Hamilton Depression Rating Scale; IES, Impact of Event Scale*.

### Diagnosis and sleep disturbances score computation

The diagnosis of PTSD was established according to the DSM-IV criteria (55). The Structured Clinical Interview for DSM-IV Axis I Disorders [SCID-I (56)] formed the basis for diagnostic assessments and was carried out by a psychiatrist not otherwise engaged in the study and blind to the experimental conditions of the participants. Further diagnostic interviews to evaluate symptoms and functional assessment included the Hamilton Anxiety Rating Scale [HAM-A (57)], the Hamilton Depression Rating Scale [HAM-D (58)], and the Impact of Event Scale (IES), evaluating the amount of intrusions and avoidance during the last week related to a past stressful event (59).

In order to quantify the amount of sleep disturbances related to insomnia and nightmares, we computed a unique Sleep Disturbances Score (SDS) by adding up the single scores of six different items taken from the IES (items 4 and 6, insomnia and trauma-related dreams), HAM-A (item 4, insomnia and nightmares), and HAM-D (items 4, 5, and 6, evening, night, and morning insomnia). Hence, SDS could range from 0 to 18, with higher scores representing a higher amount of insomnia/nightmares. Cronbach’s alpha computed on the six items was 0.82, showing good internal consistency. SDS showed high positive correlations with other clinical measures, namely a Pearson’s correlation coefficient *r* of 0.69 with IES, 0.76 with HAM-D, and 0.82 with HAM-A (all of which significant at *p* < 0.0001 after correction for multiple comparisons).

### MRI and voxel-based morphometry

Magnetic resonance imaging scanning was performed on a GE Signa 1.5 T Scanner (GE, Milwaukee, WI, USA) about 37 ± 19 months from index trauma. 3D MPRAGE T1-weighted axial images were acquired (TR = 26 ms, TE = 7 ms, FA = 35°, FOV = 220 mm × 220 mm × 149 mm, voxel size = 0.86 mm × 0.86 mm × 1.2 mm). VBM was used to pre-process and analyze MRI images using the VBM toolbox (applying standard routines and default parameters), implemented in SPM8 running under MATLAB 7.1 (Mathworks, Natick, MA, USA). To improve the accuracy of normalization and segmentation steps, data pre-processing started with manual reorienting of each subject’s dataset. Images were bias field corrected, registered using both linear (12 parameter affine) and non-linear (warping) transformations, and tissue classified, resulting in an increased quality segmentation of GM, white matter, and cerebrospinal fluid. Images were normalized to the T1 MNI template and resampled to an isotropic voxel size of 1 mm × 1 mm × 1 mm. GM tissue maps were then modulated to compensate for non-linear warping, allowing to test hypotheses about effects of relative volumes corrected for different brain sizes (60). Finally, modulated GM images were smoothed with a 12 mm (FWHM) isotropic Gaussian kernel and absolute thresholded for masking at 0.1, to exclude the influence of any remaining non-GM tissue.

### SPECT and symptom-provocation procedure

Subjects were investigated during an individualized autobiographical script-driven symptom-provocation procedure according to the method proposed by Lang et al. (61) and reported elsewhere (62). The script was read by a research assistant and recorded on tape and then presented to each subject by using earphones. After the tape had run for 15 s, 1000 MBq (27.0 mCi) of ^99m^Tc-HMPAO (Ceretec1; Amersham International plc, UK) was injected i.v. The script duration was 1′30″ and, subsequently, the subjects were asked to recall the event in their mind for one more minute. Subjects were brought to the SPECT camera 20 min later. SPECT images were acquired by a three-headed gamma camera (TRIAD XLT20; Trionix Research Laboratory Inc., USA) equipped with low-energy ultrahigh-resolution collimators, implementing previously described acquisition and reconstruction parameters (63). Both acquisition and reconstruction were performed in 128 × 128 matrices with a pixel size of 2.22 mm × 2.22 mm. Image pre-processing and analyses were performed with SPM8. All images were normalized to the SPECT MNI template by a bilinear interpolation method and smoothed with a Gaussian kernel filter of 12 mm (FWHM). GM threshold was set at 0.8, and normalization of global CBF to 50 was performed by the ANCOVA model.

### Statistical analyses

Our main analyses were run on the whole sample (i.e., irrespective of PTSD diagnosis) by creating two different design matrices, one for each dataset (i.e., MRI/SPECT; *n* = 37 each). Correlations between GM volume (GMV)/regional cerebral blood flow (rCBF) and the SDS were computed across the whole brain on a voxel-by-voxel basis by means of positive/negative correlations using the “multiple regression” model. These analyses were then complemented by running the same models in the two groups separately (PTSD and non-PTSD). Nuisance variables were introduced as follows: sex, age, and time elapsed from trauma in all analyses; PTSD diagnosis in the whole sample analyses; and intracranial volume in the VBM analyses. Given their strong correlations with SDS, anxiety and depression scores were not inserted as covariates in our main analyses (and note that SDS was computed also on the basis of some HAM-A and HAM-D items). However, when analyses were rerun by inserting such scores as covariates of no interest, we replicated our results, although with slightly reduced significance values. First, to specifically assess the impact of sleep disturbances on limbic structures, a volume-of-interest (VOI) analysis based on *a priori* hypotheses (cf. Introduction) was performed by extracting left and right amygdalae, hippocampi, and anterior cingulate cortices from the Automated Anatomical Labeling atlas [AAL (64)]. Here, a height threshold of *p* < 0.01 was chosen and then small-volume correction [FWE-corrected, *p* < 0.05 (65)] was applied within the collective volume of the six VOIs for each contrast of interest. Second, to further inspect the effects of sleep disturbances on the whole brain, exploratory analyses were run in each model by choosing a height threshold of *p* < 0.005 (uncorrected at cluster level) excluding clusters smaller than 64 (4 × 4 × 4) or 125 (5 × 5 × 5) contiguous voxels (for VBM and SPECT, respectively), based on the calculation of the partial volume effect resulting from the spatial resolution of the imaging systems, and the spatial smoothing applied. Neural structures were identified on the basis of the AAL.

## Results

The VOI analysis (Table 2) showed that sleep disturbances were associated with significant GMV reductions in bilateral amygdalae, hippocampi, and the right ACC in the whole sample and in the PTSD group (but only in the right amygdala in the non-PTSD group) and with significantly reduced rCBF in bilateral ACC in the whole sample and in the PTSD group (but not in the non-PTSD group).

**Table 2:** **Results of the volume-of-interest (VOI) analysis separately for groups, contrasts, and volumes**.

Contrast	VOI	ALL			PTSD			Non-PTSD		
		MNI	*t*	*p*	MNI	*t*	*p*	MNI	*t*	*p*
VBM SDS−	L amy	−30 −3 −22	3.43	0.013	−30 −3 −19	3.17	0.045	–	–	–
	R amy	35 −2 −24	4.82	0.001	34 −1 −22	5.23	0.002	28 4 −29	3.85	0.023
	L hippo	−32 −6 −21	3.52	0.037	−34 −10 −16	3.31	0.057	–	–	–
	R hippo	35 −4 −22	4.60	0.003	35 −4 −22	4.61	0.016	–	–	–
	L ACC	–	–	–	–	–	–	–	–	–
	R ACC	15 25 21	4.31	0.007	11 25 17	5.57	0.004	–	–	–
SPECT SDS−	L amy	–	–	–	–	–	–	–	–	–
	R amy	–	–	–	–	–	–	–	–	–
	L hippo	–	–	–	–	–	–	–	–	–
	R hippo	–	–	–	–	–	–	–	–	–
	L ACC	2 42 14	3.71	0.025	0 42 16	4.90	0.015	–	–	–
	R ACC	4 42 12	3.66	0.024	8 40 10	4.86	0.013	–	–	–
SPECT SDS+	L amy	–	–	–	–	–	–	–	–	–
	R amy	–	–	–	–	–	–	–	–	–
	L hippo	–	–	–	–	–	–	–	–	–
	R hippo	–	–	–	–	–	–	–	–	–
	L ACC	–	–	–	–	–	–	–	–	–
	R ACC	–	–	–	–	–	–	–	–	–

*ALL, whole sample (*n* = 37); SDS+/−, positive/negative correlations with the sleep disturbances score; L/R, left/right; amy, amygdala; hippo, hippocampus; ACC, anterior cingulate cortex; MNI, Montreal Neurological Institute coordinates (*x*, *y*, *z*); *t*, Student’s *t*-statistic; *p*, small-volume corrected significance at peaks (FWE-corrected)*.

**Figure 1:**
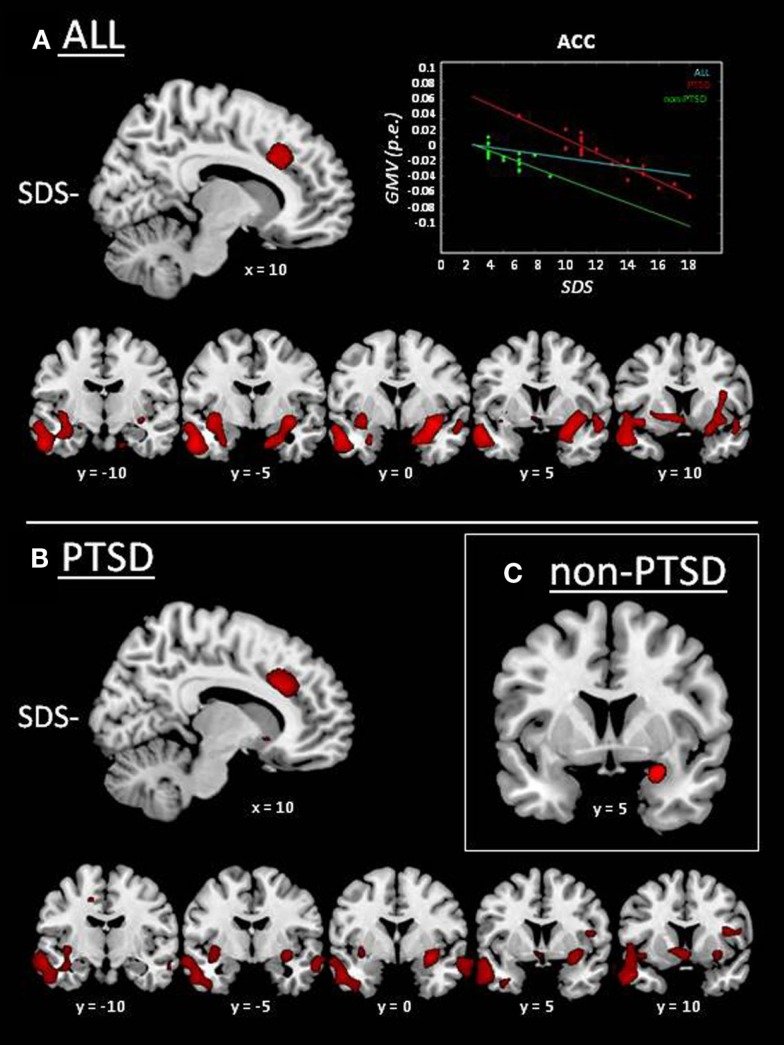
**Whole-brain results of VBM analyses on MRI data: correlation between GMV and sleep-disturbances score (SDS; cf. Table 3)**. GMV reductions associated with higher sleep disturbances (SDS−) are displayed in red. **(A)** Whole group of subjects (i.e., irrespective of PTSD diagnosis; *n* = 37). The scatter plot displays GMV as a function of SDS in the whole group (cyan line; *r* = −0.51; *p* = 0.001), and separately for PTSD (red line/diamonds; *r* = −0.94; *p* < 0.001) and non-PTSD (green line/dots; *r* = −0.73; *p* = 0.001), expressed as parameter estimates (p.e.; values extracted at peak in the anterior cingulate cortex). **(B)** PTSD group. **(C)** Non-PTSD group.

Whole-brain VBM analysis in the whole sample showed significantly reduced GMV with sleep disturbances in the right ACC, bilateral insular cortex, the complex amygdala/hippocampus/parahippocampal cortex bilaterally extending into the temporal poles, left striatum, and the right dorsolateral prefrontal cortex (Figure 1A; Table 3). No significant positive correlation was found between sleep disturbances and GMV. This pattern of results was substantially confirmed in the PTSD group (Figure 1B; see Table 3 for details), whereas in the non-PTSD group significantly reduced GMV with SDS could only be found in the right amygdala (Figure 1C).

**Table 3:** **Whole-brain structural and functional neuroimaging results in the whole sample (ALL; *n* = 37) and in the PTSD group**.

Contrast	Region	BA	ALL				PTSD			
			MNI	*K*	*t*	*p*	MNI	*K*	*t*	*p*
VBM SDS−	R anterior cingulate cortex	24/32	14 24 20	1897	4.35	<0.001	12 25 17	2796	5.70	<0.001
	R amygdala	–	23 −3 −33	10140	3.39	0.001	29 0 −26	2527	3.51	0.003
	L amygdala	–	−23 0 −35	16363	2.76	0.003	–	–	–	–
	R hippocampus	–	27 −7 −31	10140	2.81	0.003	–	–	–	–
	L hippocampus	–	−31 −12 −30	16363	2.96	0.003	−35 −13 −30	18728	3.08	0.003
	R anterior insula	13	42 17 −26	10140	3.32	0.001	41 11 4	800	4.06	0.001
	L anterior insula	13	−42 13 −26	16363	3.56	0.001	−40 12 −23	18728	3.96	0.001
	R posterior insula	13	38 −2 −19	10140	3.96	<0.001	38 −1 −21	2527	4.48	<0.001
	L posterior insula	13	−36 −6 −19	10140	3.63	0.001	−35 −8 −16	18728	3.66	0.001
	R parahippocampal cortex	28/36	20 −7 −41	10140	3.49	0.001	–	–	–	–
	L parahippocampal cortex	28/36	−27 −6 −39	16363	2.94	0.003	–	–	–	–
	R dorsolateral prefrontal cortex	10/46	35 50 3	806	4.19	<0.001	–	–	–	–
	R orbitofrontal cortex	11	–	–	–	–	20 36 −23	1135	3.84	0.001
	L postcentral gyrus	1/2/3	–	–	–	–	−46 −23 33	1031	4.67	<0.001
	L striatum	–	−12 11 −18	1175	3.38	0.001	0 12 −20	1471	4.51	<0.001
SPECT SDS+	L/R dorsal midbrain	–	2 −24 −18	459	4.39	<0.001	4 −28 −22	319	4.81	<0.001
	R precuneus	7	12 −52 32	302	3.37	0.001	10 −54 30	590	3.35	0.003
	L precuneus	7	−12 −54 26	302	3.59	0.001	−12 −52 28	590	5.00	<0.001
	R posterior insula	13	34 −22 0	562	4.30	<0.001	–	–	–	–
	L posterior insula	13	–	–	–	–	−42 −30 6	316	3.52	0.002
SPECT SDS−	L/R anterior cingulate cortex	24/32	2 42 14	700	3.71	<0.001	042 16	2651	4.90	<0.001
	R dorsolateral prefrontal cortex	9	–	–	–	–	24 46 24	2651	4.69	<0.001
	L orbitofrontal cortex	11	–	–	–	–	−16 48 −18	2651	5.83	<0.001

*SDS+/−, positive/negative correlations with the sleep disturbances score; L/R, left/right; BA, Brodmann area; MNI, Montreal Neurological Institute coordinates (*x*, *y*, *z*); *K*, cluster size; *t*, Student’s *t*-statistic; *p*, statistical significance at peaks (uncorrected at cluster-level)*.

**Figure 2:**
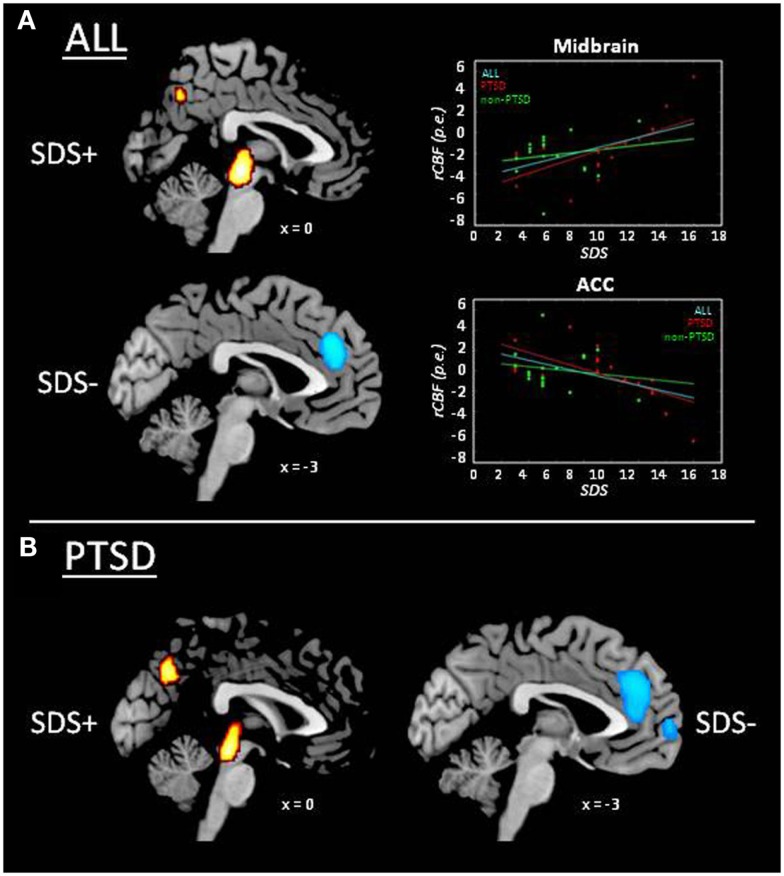
**Whole-brain results of the analysis on SPECT data: correlation between rCBF and sleep disturbances score (SDS; cf. Table 3)**. rCBF increase associated with higher sleep disturbances (SDS+) is displayed in red/yellow and rCBF decrease with higher sleep disturbances (SDS−) in light blue. **(A)** Whole group of subjects (i.e., irrespective of PTSD diagnosis; *n* = 37). Scatter plots display rCBF as a function of SDS in the whole group (cyan line; *r* = ±0.51; *p* = 0.001), and separately for PTSD (red line/diamonds; *r* = ±0.68; *p* = 0.001) and non-PTSD (green line/dots; *r* = ±0.17; *p* = 0.511), expressed as parameter estimates (p.e.; values extracted at peaks in the midbrain and anterior cingulate cortex). **(B)** PTSD group.

**Figure 3:**
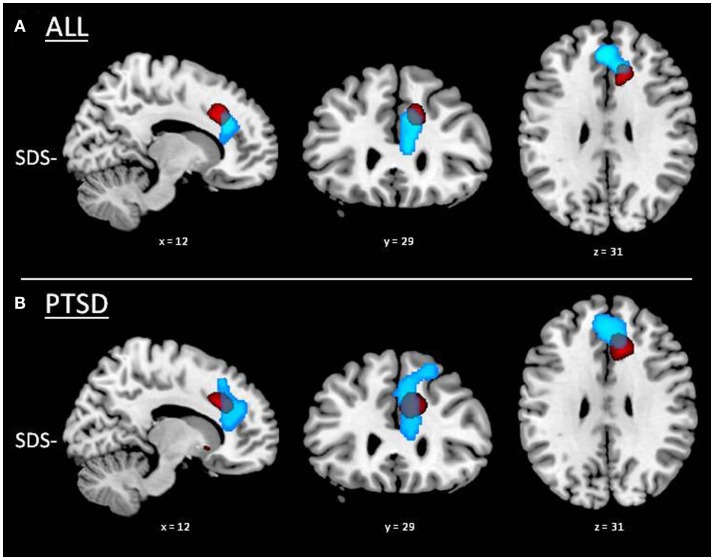
**Partial overlap between VBM and SPECT results in the anterior cingulate cortex**. GMV reductions associated with higher sleep disturbances (SDS−) are displayed in red and rCBF decrease with higher sleep disturbances (SDS−) in light blue. **(A)** Whole group of subjects (i.e., irrespective of PTSD diagnosis; *n* = 37). **(B)** PTSD group.

Whole-brain functional results in the whole sample showed significantly increased rCBF with sleep disturbances in bilateral midbrain, right posterior insular cortex, and bilateral precuneus (Figure 2A; Table 3). A negative correlation was also found, showing significant decreased rCBF with sleep disturbances in bilateral ACC. This latter cluster showed a partial overlap with the ACC cluster found in the VBM analysis (Figure 3), indicating that sleep disturbances are associated with decreased GMV and rCBF in the same region. Again, this pattern of results was substantially confirmed in the PTSD group (Figure 2B), the most notable exception being that the cluster located in bilateral ACC extended dorsally into the right dorsolateral prefrontal cortex and ventrally into the left orbitofrontal cortex (cf. Table 3). No significant rCBF alterations were found associated with SDS in the non-PTSD group.

## Discussion

The present study was aimed at investigating brain alterations related to sleep disturbances in PTSD by using VBM and SPECT. Structural neuroimaging showed reduced GMV with higher insomnia/nightmares symptoms in the amygdala, hippocampus, ACC, and insula. Functional neuroimaging showed that a higher amount of sleep disturbances was associated with decreased rCBF in the ACC and increased rCBF in the midbrain, precuneus, and insula. Before discussing our findings in detail, two general considerations ought to be made.

First, our VBM results show that brain structures on which sleep disturbances impact (amygdala, hippocampus, ACC) largely overlap with regions well known in the literature to be PTSD-sensitive [cf. Ref. (24, 25)]. Importantly, our results show that the effects found in the whole sample are primarily driven by the PTSD group. This indicates that PTSD and insomnia/nightmares share a substantial neurobiological substrate, supporting the existence of a strong link between the two (2, 3, 8), or even the idea that sleep disturbances might constitute the core characteristic of PTSD (66, 67).

Second, our SPECT results show that the amount of insomnia/nightmares is associated with a specific pattern of brain alterations in a subset of regions known to be modulated during REM sleep. In fact, previous studies have shown that REM sleep is associated with a relative increase of activity in the midbrain, ACC, and insula and a relative decrease of activity in the precuneus (68–70). Hence, our results show that under emotional stress (i.e., during symptom provocation), such regions show sleep disturbances-related functional alterations, either consistent or inconsistent with REM-sleep modulations.

Several studies on PTSD report either increased amygdalar activity (31, 32) or reduced amygdalar volume (71–73). In the context of PTSD, the amygdala has been related to fear conditioning and hyperarousal, this latter is also a putative determinant of insomnia (74, 75). Together with the hippocampus and ACC, the amygdala is thought to be implicated in reexperiencing phenomena such as intrusions and nightmares. The amygdala is also considered a key structure in two neurobiological models concerning sleep-specific mechanisms underlying PTSD and the generation of nightmares. According to the former, a prolonged hyperactivity of the amygdala (as in PTSD) might increase the generation of nightmares and alter neural activity in the brain stem and forebrain sleep-regulating centers (52). According to the latter, the amygdala (together with the hippocampus and ACC) plays a key role in the processing of fear consolidation, fear extinction, and emotional distress, processes that underlie nightmare generation and are capable of affecting activity in the brain stem and hypothalamic autonomic centers (53). To the best of our knowledge, the present study is the first to report structural amygdalar alterations in association with sleep disturbances, also in traumatized controls who do not develop PTSD.

Previous investigations also consistently report decreased hippocampal volumes in PTSD (26–29, 76–78), where it is thought to play a major role in the consolidation of traumatic memories. Besides, some studies showed that reduced volume in this structure is associated with presence and severity of insomnia [Ref. (36–39); but see Ref. (35, 40, 41)]. One possible explanation for this inconsistency might arise from the idea that hippocampal volume reductions are associated with sleep disturbances only in combination with trauma exposure. Traumatic stress is known to heavily impact on the hippocampus (79, 80), yet it has been suggested that this might be mediated by sleep disturbances (81), similarly to how trauma type, severity, and cumulative load as well as personality traits are known to modulate the outcome of trauma itself. Although most studies on insomnia have carefully excluded subjects suffering from other comorbid conditions, they might have overlooked essential information on possible trauma exposure. This latter should not necessarily result in an overt psychiatric condition but might nonetheless impact on hippocampal volume in the presence of insomnia and nightmares, both strongly associated with hyperarousal and considered stressors by themselves (8). One putative mechanism of the interaction between trauma and sleep disturbances might reside in the role played by the hippocampus in triggering trauma-related memories, which might foster nightmares and insomnia (53), feeding, in turn, the reexperiencing of trauma, hence maintaining and/or exacerbating PTSD symptoms.

Our results indicate that sleep disturbances have a particularly serious impact on the ACC, by altering both its structure and function (reduced GMV and rCBF). Several previous structural neuroimaging studies on PTSD consistently report volume reductions in the ACC (72, 82, 83). Conversely, one recent study reported larger volumes in the ACC in insomniacs and a correlation between insomnia severity and GMV (44). The ACC is a key element of the neurocognitive model of nightmares generation, playing a fundamental role in the modulation of amygdalar activity (53). Thus, our present findings of structural and functional alterations in the ACC might reflect an impaired control over limbic activity during wakefulness and sleep, possibly leading to REM-sleep disruption (69, 70), hyperarousal, and/or reexperiencing (i.e., insomnia/nightmares). The ACC underlies different functions, ascribable not only to emotional regulation but also to executive functions (84). Therefore, an impairment of this structure might also account for the daytime cognitive disturbances commonly reported by insomniacs, such as reduced alertness, performance, and concentration capacity, sleepiness/fatigue, and higher proneness to errors/accidents at work (85, 86).

Our results show structural and functional alterations in the insular cortex as related to sleep disturbances. More specifically, we found decreased GMV in anterior and posterior insula and increased rCBF in posterior insula. Structural and functional alterations in the insula have been reported by previous neuroimaging studies on PTSD (87–89). Furthermore, insomnia severity was associated with reduced rCBF (47), and functional connectivity between insula and amygdala was found to be either reduced or enhanced in insomniacs (48, 49). The insular cortex is functionally organized along a posterior–anterior gradient, the posterior portion being more related to interoception and emotion-related bodily sensation and the anterior to emotional awareness and subjective feelings (90). Together with the ACC, the insula is also part of the salience network, which integrates highly processed sensory data with visceral, autonomic, and emotional information (91). Insomniacs show a greater involvement of anterior insula within the salience network associated with negative feelings (92). Hence, our finding of GMV reductions in the insula with higher sleep disturbances might reflect reduced interoceptive/emotional processing due to chronic insomnia while increased activity in posterior insula might be associated with enhanced pain perception or negative bodily sensations related to the reexperiencing of trauma, which in turn might contribute to foster insomnia and/or nightmares generation.

Previous works on functional correlates of REM sleep have shown that the precuneus is relatively deactivated during this state (69). Conversely, our results show that (under emotional stress) activity in the precuneus increases with a higher amount of insomnia/nightmares. A few previous studies have shown a link between sleep disturbances and precuneus. There, GMV was found to inversely correlate with insomnia severity (40) while greater activity was associated with eveningness, a trait associated with a higher presence of nightmares, insomnia, PTSD symptoms, and emotional dysregulation (93). The precuneus is involved in a number of cognitive functions such as episodic and autobiographical memory (94), consciousness and self-reflection (95, 96), visuospatial representation, and mental imagery, the so-called mind’s eye (97, 98). Hence, its involvement in trauma reexperiencing is not surprising. However, our results indicate that such reexperiencing probably occurs also while sleeping, most likely in form of nightmares. Anyway, a relative increase of activity in this structure while sleeping might create a functional imbalance capable of disrupting REM sleep (69), possibly fostering insomnia.

Finally, our results show that sleep disturbances are associated with increased rCBF in bilateral dorsal midbrain, a region including the ascending reticular activating system, a well-known sleep-regulating center. Interestingly, increased activity in this region of the brain stem has been reported in PTSD subjects during wakefulness and REM sleep (51) and in association with eveningness in combat veterans (93). Moreover, increased activity in this region was found during REM sleep in depressed patients who typically suffer from insomnia (99). One possible explanation for our present finding is that the increased activity in this region reflects enhanced physiological arousal associated with trauma reliving, which in turn might favor the generation of nightmares and insomnia during sleep (74, 75), possibly as a consequence of increased activity in the amygdala and/or reduced inhibitory control of the ACC. Alternatively, such activity might constitute an attempt to counterbalance insomnia effect and to stabilize sleep by enhancing activity in the ascending reticular activating system.

The present study has some limitations that need to be acknowledged. (1) We used only subjective measures of sleep disturbances. Since subjective measures might suffer from specific disadvantages (e.g., exaggeration, social desirability, and memory lapses), our results need to be corroborated by further studies using objective measures. (2) Functional alterations were determined during symptom provocation. This might make less straightforward the interpretation to what extent our SPECT findings are related to symptom provocation or sleep disturbances. Hence, our results need to be confirmed by future studies using functional neuroimaging at rest or while sleeping. (3) Our subjects showed moderate-to-high scores in anxiety and depression. Although these were subclinical, they might have impacted on our results. However, as we describe in the Section “Statistical Analyses,” our results were substantially replicated once these scores were factored out. (4) Three subjects were under psychopharmacological treatment while participating in our study, with a possible impact on our findings. However, it should be considered that these subjects represented a very small fraction (less than one-tenth of the sample) and that they were almost equally distributed in the two groups (two PTSD, one non-PTSD).

The present study also has some qualifying points that deserve to be pointed out. (1) We used a relatively large sample of subjects. (2) Structural and functional imaging measures were collected in the same sample. (3) We recruited traumatized controls (instead of healthy subjects) against which to compare PTSD patients. (4) We inserted PTSD diagnosis as a nuisance variable in correlational analyses with sleep disturbances.

## Conclusion

In conclusion, we found that sleep disturbances are associated with GMV loss in limbic, PTSD-sensitive structures and with functional alterations in regions implicated in REM-sleep control. These findings support the existence of a neurobiological link between PTSD and sleep disturbances. Whether this implies that restorative sleep helps in protecting against GM loss (and possibly against higher vulnerability to PTSD symptoms) and/or in enhancing the effectiveness of stress response is yet to be determined. In any case, we recommend that future investigations on PTSD should take into account the influence of sleep disturbances.

## Conflict of Interest Statement

The authors declare that the research was conducted in the absence of any commercial or financial relationships that could be construed as a potential conflict of interest.
